# Crowdfunding abortion: an exploratory thematic analysis of fundraising for a stigmatized medical procedure

**DOI:** 10.1186/s12905-020-00938-2

**Published:** 2020-05-04

**Authors:** Marco Antonio Zenone, Jeremy Snyder

**Affiliations:** Simon Fraser University, Blusson Hall - 8888 University Dr, Burnaby, BC V5A 1S6 Canada

**Keywords:** Medical Crowdfunding, Abortion, GoFundMe, Stigma, Women’s health

## Abstract

**Background:**

Medical crowdfunding is the process of using a crowdfunding platform to raise funds for medical treatment and associated expenses, such as missing work or transportation costs to access care. This type of crowdfunding has become increasingly popular, and is an effective tool to raise financing for medical treatment in the absence of insurance. However, it is accompanied by questions of which diseases or treatments are viewed as worthy to fund and which do not fit the criteria of worthiness. In the context of an abortion, a legitimate and important medical procedure, there is a lack of research that determines if campaigners can successfully utilize GoFundMe to pay for abortions and abortion related services and costs given the social stigma around this procedure. Here, we explore the outcomes of crowdfunding campaigns for stigmatized needs and conditions by examining campaigns related to abortion.

**Methods:**

A total of 211 campaigns that utilized the term “abortion” were retrieved on the medical-section of the GoFundMe crowdfunding platform. These results were thematically analyzed by each author and two distinctive categories were identified to group the campaigns.

**Results:**

The categories of campaigns using the term “abortion” were: campaigns seeking funds to access abortion related services (*n* = 84) and campaigns using the choice not to terminate pregnancy or the harms of abortion as a reason to give (*n* = 127). The number of donors, number of Facebook shares, campaign location, funding requested, funding pledged, campaign creation date, relation between the recipient and campaigner, and proposed use for the funds were recorded for each included campaign.

**Conclusions:**

This study suggests that certain conditions or diseases may be less successful in medical crowdfunding based on perceived features of worthiness, such as in the case of abortion. In the categories we identified, campaigns seeking funds to access abortion-related services were less successful than campaigns using choosing not to terminate a pregnancy or the harms of abortion as a reason to give. This is an area of concern in medical crowdfunding – that certain medical needs will not be funded equitably.

## Background

Medical crowdfunding is the process of using a crowdfunding platform to raise financing for medical treatment and related costs, including financial support if the intended recipient is unable to work due to theirs or a family member’s condition [1]. This form of crowdfunding predominately takes place on the crowdfunding platform GoFundMe – which has been aggressively expanding and acquiring smaller crowdfunding platforms that raise money for similar charitable causes, such as YouCaring and CrowdRise [2–4]. GoFundMe has seen substantial increases in use of their platform – 8000 campaigns were active in 2011 that raised approximately $1.6 million, increasing in 2014 to 600,000 active campaigns and approximately $150 million raised [5]. The most recent figures released by GoFundMe claim that in 2018, $750 million was raised for medical purposes [6]. Studies indicate that as many as 22% of Americans have contributed to at least one crowdfunding campaign, with approximately 3% having hosted their own campaign [7].

The popularity of medical crowdfunding can be attributed to filling a gap in healthcare financing, particularly in contexts where universal healthcare or comprehensive medical insurance is not present or accessible [5, 8, 9]. Crowdfunding platforms such as GoFundMe allow users to raise funds for medical costs that they otherwise could not have afforded or would have struggled to afford [8, 9]. Common medical reasons that users campaign for are cancer treatment, experimental treatments for various diseases, and elective treatments not covered by health insurance [7]. There are regularly news stories that report on such examples, such as a Canadian campaign in 2018 that raised approximately $108,000 dollars to fund a German experimental cancer intervention [10].

While the success of this and similar campaigns has promoted access to medical care and experimental interventions, researchers have raised ethical concerns over which diseases or conditions get financed, and which do not [1, 5, 9, 11, 12]. It has been suggested that diseases or conditions which are stigmatized – such as fundraising for an abortion procedure or human immunodeficiency virus / acquired immunodeficiency syndrome treatment (HIV/AIDS) – may fare poorly against other conditions due to being perceived as less worthy and deserving of support [5, 9]. The policies of the platform hosting the campaign, which dictate in many cases what can be campaigned for, additionally may contribute to limitations on the successful financing of stigmatized diseases and conditions if they violate community standards of acceptable medical treatments [13]. However, the variable crowdfunding success of stigmatized conditions has been largely unexplored and there is a lack of empirical research to support these concerns.

In this paper, we seek to explore the outcome of crowdfunding campaigns for a single stigmatized need by examining campaigns related to abortion on GoFundMe, adding to the growing but underdeveloped literature on crowdfunding for stigmatized needs and conditions. While many women have legal abortions based on their specific contextual needs, the action of completing an abortion is often subject to intense scrutiny and implied blame on the person having an abortion due to prevalent negative societal attitudes [14, 15]. Recent surveys conducted in the United States found approximately 48% of Americans believe having an abortion is morally wrong and approximately 38% believe it should be illegal [16]. These negative perceptions of abortions were previously reflected in GoFundMe policy, as campaigners were not able to raise funds for abortion when a policy banning this type of campaign was briefly put in place in 2014 [13]. This policy has since been reversed, but campaigns seeking funds for abortion may still face significant challenges and thus reaffirm that social stigma influences online fundraising [17].

## Methods

The word “abortion” was searched under the “medical” category using GoFundMe’s internal search engine in May 2018. This search returned 286 campaigns. Each campaign was reviewed by the authors to determine eligibility for inclusion in this research study. Campaigns were considered to be eligible for inclusion if they were specifically fundraising for an abortion, abortion-related services, or if abortion was in part used as the rationale to encourage donating to a campaign. Common reasons for exclusion were that the campaigns included the term “abortion” but it was not a significant component of the rationale for giving (*n* = 55), the campaign was clearly fake (*n* = 5), the term “abortion” was used to refer to a miscarriage or an unsuccessful pregnancy (*n* = 12), or the campaign did not have enough detail to determine if abortion was part of the rationale for giving (*n* = 3). The authors agreed on and noted the rationale for all excluded campaigns. After applying these criteria, the total numbers of included campaigns was 211.

The number of donors, number of Facebook shares, campaign location, funding requested, funding pledged, campaign creation date, relation between the recipient and campaigner, and proposed use for the funds were recorded for each included campaign. The campaign text was independently analyzed by each author and common themes were noted. The authors subsequently met to discuss these themes and agreed to sort the campaigns into two distinct categories based on how the term “abortion” was used: (1) Campaigns seeking funds to access abortion-related services and (2) Campaigns using the choice not to terminate a pregnancy or the harms of abortion as a reason to give. The first category includes all cases where the goal of the campaign is to fund a specific abortion procedure or raise general funds for other women to have an abortion due to medical reasons, an unwanted pregnancy, indirect costs related to the abortion such as travel or missed income during the procedure, or other hardships. The second category includes all campaigns where the organizer has specifically stated they are not having an abortion despite experiencing a form of hardship, recovering from forced/unwanted abortions, or using an ideological argument to prevent unwanted pregnancies as a rationale to give. After each campaign was initially categorized by the first author, both authors met to confirm the categorization of each campaign. At this time, the authors agreed to additional sub-categories for each campaign.

## Results

The 211 crowdfunding campaigns predominately originated in the United States (*n* = 193), with other campaigns located in the United Kingdom (*n* = 6), Canada (*n* = 5), Australia (*n* = 5) and Norway (*n* = 2). The campaigns retrieved were posted on the GoFundMe platform between June 2011 and May 2018. The campaigns raised a total of $424,710.74 United States dollars (USD) from 5114 donors and were shared on Facebook 40,722 times.

### Campaigns seeking funds to access abortion related services

In the first category, 84 campaigns were identified (Table 1). These campaigns had an average of 4 donors, $138.82 pledged and $874.16 requested. Only 21 of the 84 (25.0%) campaigns received a donation (63 receiving no contributions). These campaigns were shared an average of 13.5 times on Facebook. The organizers of these campaigns were mainly a pregnant person who was the intended recipient of the donations (*n* = 44), followed closely by a friend of the recipient (*n* = 16), the sexual partner of the recipient (*n* = 13), the recipient’s family members (*n* = 4), pro-choice organizations (*n* = 2), and unknown relationships (*n* = 5).

**Table 1:** Campaigns seeking funds to access abortion related services summary

Thematic areas	Number of campaigns	Average # of donors	Average # of Facebook shares	Average amount raised	Average amount requested	Amount w/ no donations
Abortion due to non-medical hardship/circumstance:						
- Lack of money/unready	25	2.1	8.0	$83.80	$866.51	21 (84.0%)
- Age	23	5.0	4.7	$111.68	$803.60	20 (8.70%)
- Rape	15	4.5	14.1	$120.34	$788.00	11 (73.3%)
- Family & father pressure/factors	3	1	0	$156.67	$1298.00	2 (66.7%)
Abortion fund/pro-choice justification	5	4.8	14	$280.97	$849.74	2 (40.0%)
Abortion due to medical risks (mother)	3	7.3	104	$200.00	$690.00	1 (33.3%)
Abortion due to defect/critical illness (baby)	2	15.5	101.5	$885.00	$2100.00	0 (0.0%)
Unwanted pregnancy/unknown reasons	8	2.9	4.1	$118.41	$881.42	6 (75.0%)
**Total**	**84**	**4**	**13.5**	**$138.82**	**$874.16**	**63 (75.0%)**

Several thematic areas were identified under the overarching category of campaigns seeking funds to access abortion-related services. The first thematic area was experiencing a non-medical hardship related to an unwanted pregnancy that served as a rationale for seeking abortion services (*n* = 66). The first form of non-medical hardship was the pregnant woman not having the financial means to support the baby or themselves (*n* = 25). These campaigns were predominately for women experiencing various forms of poverty or low-income who reported being unable to afford the cost of raising a child. The next form of non-medical hardship were campaigners stating they are too young to care for a child and thus in need of an abortion (*n* = 23). For instance, in one campaign a 14-year-old boy pleaded for assistance to help fund an abortion and explained the situation to be “life changing” if they cannot raise enough funds; as was typical of campaigns in this category, this campaign did not raise any money and was not shared on any social media platform [18]. Other forms of hardship included pregnancy as the result of a sexual assault (*n* = 15). These included a young woman who was sexually assaulted and wanted funds to abort the pregnancy as “the fetus growing is a continuation of his violation”; again, this campaign did not raise any money or result in any Facebook shares [19]. A final form of hardship in this area were cases where the pregnant woman’s family or sexual partner was abusive or applying pressure to abort the pregnancy (*n* = 3).

The second thematic area identified included campaigns that were not raising money for abortion services for a specific recipient, but rather to support abortion funds that either provided or subsidized abortion services for women who have financial or social barriers to accessing them (*n* = 5). These were compromised of various specific pro-choice organizations or individuals making a political statement as justifications for funds. For example, a Michigan campaign was raising money for the Fountain Street Choice Fund that “provides monies for women who find it a financial burden to come up with monies for the abortion services” [20].

The third thematic area identified included campaigns where the campaigner was seeking financial support for abortion due to medical necessity (*n* = 3). These campaigns involve situations where the pregnant recipient cannot safely deliver the baby due to various medical complications or conditions and thus is given the option of an abortion to preserve their health. These campaigners typically express a wish to be able to bring the fetus to term but may choose not to do so for their own health and safety. For example, a North Carolina woman writes “I am currently, and will likely always be, high risk when pregnant” and further elaborates that she often experiences excessive bleeding, and this poses significant challenges to her health when pregnant [21].

The next theme identified were campaigns where the campaigner was seeking financial support to abort the pregnancy due to knowledge of the fetus having a severe defect or critical illness (*n* = 2). An example of this type of campaign is where a woman is seeking funds to have an abortion where the fetus was missing/developing abnormal organs [22].

The last group identified consisted of campaigns where an unwanted pregnancy was reported without meeting criteria to be included in the thematic areas above, yielding 8 campaigns. These included campaigns where not enough detail was offered to make a positive determination of why termination of a pregnancy was desired.

### Campaigns using the choice not to terminate a pregnancy or the harms of abortion as a reason to give

In the second category, 127 campaigns were identified (Table 2). These campaigns had an average of 37.6 donors, $3252.36 raised, $27,350.03 requested, with 93 of the 127 (73.2%) campaigns having received a donation (34 receiving no contributions). These campaigns were shared an average of 312.1 times on Facebook. The organizers of these campaigns were mainly the person declining an abortion or their significant other (*n* = 81), followed closely by a friend (*n* = 20), family members (*n* = 16), pro-life organization (*n* = 8), pastor (*n* = 1), or journalist (*n* = 1).

**Table 2:** Campaigns Using the Choice Not to Terminate a Pregnancy or the Harms of Abortion as a Reason to Give

Thematic areas	Number of Campaigns	Average # of donors	Average # of Facebook shares	Average amount raised	Average amount requested	Amount w/ no donations
Did not have abortion despite defect/critical illness (baby)	57	37.1	317.9	$3447.19	$25,907.26	5 (8.8%)
Did not have abortion despite illness/risks (mom)	27	37.7	326.2	$2503.41	$38,225.54	5 (18.5%)
Preventing abortions/prolife justification/abortion as negative	18	68.1	521.9	$6756.16	$35,351.71	9 (50.0%)
Did not have abortion despite non-medical hardship/circumstance:						
- Family & father pressure/factors	6	5.8	24.2	$641.67	$29,225.93	5 (83.3%)
- Age	4	3.3	35.8	$175.00	$1750.00	3 (75.0%)
- Rape	1	265	2100	$17,606.00	$20,000.00	0 (0.0%)
- Lack of money/unready	1	1	37	$100.00	$10,000.00	0 (0.0%)
Abortion as causing trauma in need of treatment	8	9.5	80.4	$520.31	$12,395.68	4 (50.0%)
Family planning	5	5.2	49.8	$187.74	$3359.75	3 (60.0%)
**Total**	**127**	**37.6**	**312.1**	**$3252.36**	**$27,350.03**	**34 (26.8%)**

Several thematic areas were identified under the overarching category of campaigns referencing using the choice not to terminate a pregnancy or the perceived harm of abortion as the rationale for giving. The first theme identified consisted of recipients who completed a pregnancy despite being informed of a critical defect or health condition of the fetus (*n* = 57). These campaigns were mainly comprised of situations where a medical professional has offered or advised the option of abortion after revealing the fetus may have a significant birth defect or a substandard quality of life, but this suggestion was refused by the recipient. An example of this type of campaign is a Maryland-based fundraiser where a woman was informed her fetus has Pierre Robin Syndrome which results in the lower jaw and low chin not fully developing and several other defects such as hip dysplasia, fused wrists/elbows, kidney problems, and cardiac issues [23]. The organizer stated within this campaign that “we were told months before she was born that she had a slew of abnormalities but, as my wife and I are against abortion, all we could do was pray and wish for the best” [23].

The second thematic area identified included not terminating the pregnancy despite it posing significant health challenges for the recipient (*n* = 27). In these campaigns, the mother had a significant health condition such as cancer or severe complications from pregnancy but opted against having an abortion. For example, in one campaign a Texas woman was diagnosed with cancer and opted against the recommendations of her doctor to have an emergency abortion saying she “didn’t take a second to tell me no [and] there was nothing to discuss” [24].

The next thematic area specifically discussed using funds to campaign against the legality of offering abortion services (*n* = 18). These campaigns consisted of groups or persons that advocate against abortions and are using their stance to raise funds for their various causes. For example, a North Carolina campaign titled “Help Fight Abortion” reads “I have thought about this and I strongly believe that murder of an infant is cruel and horrible. I would like to help put a stop to it” [25]. This campaign attempted to raise money to prevent abortions through an unspecified initiative.

The fourth thematic area identified was choosing to not have an abortion despite experiencing a form of non-medical hardship (*n* = 12). A common form of non-medical hardship in this area discussed completing a pregnancy despite pressure from the recipient’s family or sexual partner to abort the fetus (*n* = 6). This included an Ohio campaign where “the father [of the fetus] tried to force me to have an abortion” [26]. Several campaigns described completing a pregnancy despite being in an age demographic that poses significant difficulties (*n* = 4). This group included situations where a person may be very young but still chooses to complete the pregnancy despite the difficulties of doing so. For example, a Kentucky-based campaign reads “we are both teens and we both want to prepare for the baby and give her the best life she can have” [27]. Finally, one campaign reported completing a pregnancy resulting from sexual assault (*n* = 1) and another from lack of money (*n* = 1).

The next thematic area consisted of campaigns that sought funding to repair trauma resulting from an abortion (*n* = 8). These campaigns consisted of situations where persons needed treatment after they were forced to have an abortion or the baby/person having an abortion suffered physical or mental distress resulting from an attempted or completed abortion. For example, a Washington, DC campaign was raising money for a three-year-old with facial defects and the campaign content reads “I suffer some birth defects; probably due to attempted abortion” [28].

The sixth theme identified were campaigns raising money for contraceptive or family planning services using the rationale of preventing a pregnancy – and potentially an abortion – from occurring (*n* = 5). In one of these campaigns, the campaigner sought funds for various tubal ligation surgeries or access to contraceptive services with the rationale that if the campaign were funded, she would not have to get an abortion [29].

## Discussion

### Campaign success by category

Campaigning using the choice not to terminate a pregnancy or the harms of abortion as a reason to give raised more money than campaigns seeking funds to access abortion related services. Those in the first category raised an average of $3252.36 (average request of $27,350.03) per campaign versus the latter category which averaged approximately $138.82 (average request of $874.16) per campaign. An important consideration when interpreting the above figures is the cost of raising a child significantly exceeds the cost of an abortion procedure, therefore potentially explaining the difference in campaign funding requests. Thus, campaigns in the first category raised 11.9% of their overall goals whereas campaigns to terminate a pregnancy raised 15.9% of requested funds. Those choosing not to terminate their pregnancies received support from many more people as they averaged 37.6 donors per campaign, 312.1 shares on Facebook and had 73.2% of campaigns receive at least one donation. In comparison, campaigns seeking to raise funds for abortion averaged 4 donors per campaign, 13.5 Facebook shares, and only 25.0% of campaigns receiving at least one donation. Thus, there is a stark difference in the total amount pledged and campaign reach between these two categories despite the greater percentage of the overall goal raised by campaigns seeking funds to terminate a pregnancy.

### Selected worthiness of disease & illness

These results support concerns that in crowdfunding, certain illnesses or needs are considered worthier for financial support than stigmatized diseases or needs such as abortion. In the context of this research, our findings show that campaigns to have or need an abortion are less likely to receive any financial support as compared to those who are choosing not to have an abortion or who are highlighting the harms of abortion. This view persists despite that most campaigns seeking funds for abortion reported extenuating circumstances that pose significant mental and physical risks to the mother and/or fetus such financial insecurity (*n* = 25), young age (*n* = 23), pregnancy as the result of sexual assault (*n* = 15), family issues (*n* = 3), medical risks (*n* = 3), and critical illness or deformity in the fetus (*n* = 2). Campaigns funding for abortion services may be less successful at least in part due to the stigma and culture of blame surrounding the procedure. As result, campaigners may be reluctant to promote their campaign on their social network and rely on strangers for funding. This is evidenced by the vast difference of average Facebook shares (13.5 vs. 312.1).

Those who were fundraising based on the rationale of choosing not to receive an abortion or the harms of abortion were conversely more able to take advantage of a context that allows for positive reception on social media. This included situations where a pregnancy was taken to term in cases of critical illness or disfigurement of the fetus (*n* = 57), medical risks for the mother (*n* = 27), abortion depicted as causing trauma (*n* = 8), family pressures (*n* = 6), access to family planning to prevent abortions (*n* = 5), age (*n* = 4), financial insecurity (*n* = 1), and pregnancy as the result of sexual assault (*n* = 1). This category also included specific fundraising campaigns for organizations or individuals seeking to prevent abortions on a public scale (*n* = 18). These situations and intentions may have benefitted from the perceived heroics and perseverance of the organizers.

### Concerns about medical crowdfunding

GoFundMe positions itself to the public as an effective tool to raise funds for medical treatment where financial resources are not available and thus filling an important gap in healthcare financing. For instance, on the “Start a Fundraiser” page, GoFundMe states “Get help paying for medical bills, treatments, and other healthcare expenses with medical fundraising on GoFundMe. We offer free fundraising for your needs” [6]. While this is an admirable mission there are concerns with the accuracy of this and other similar statements. Campaigns are often successful because they describe specific hardships that garner sympathy, but the platform and its donors do not necessarily equitably fund campaigns that need financial contributions. This is contradictory to the purpose of using crowdfunding platforms to raise money for healthcare financing. Those who experience an illness or situation that is stigmatized are disadvantaged in raising funds to support their treatment or healing. This is starkly displayed in the politically charged context of abortion. While the existence of platforms that crowdfund for medical care is the product of inequity, it is important to acknowledge that certain arbitrary features of worthiness still influence which campaigns are successful. This depicts that GoFundMe, even if unwillingly and unintentionally, reinforces stigmatized diseases or situations. However, the responsibility of ensuring just outcomes does not fall solely on GoFundMe and is a symptom of larger social problems.

### Future research

This research highlights one example where a specific medical treatment or condition is potentially less successful in raising financial support based on its perceived worthiness within the general population. While this example reveals substantial differences in fundraising in the context of abortion, there are other areas that need to be further explored. The motivations of donors should be explored directly, including through interviews with this group. Our findings on the differential success of abortion-related campaigns suggests that persons with needs related other stigmatized needs such as treatment for alcohol and drug dependency, mental health treatment, and HIV/AIDS are likely to struggle with using crowdfunding to address these needs. Thus, research is needed to assess if the stigma and perceived worthiness of other illnesses such as mental disorders and liver disease are reinforced through using the GoFundMe platform, to what degree, and, if so, whether differential success with medical crowdfunding can be addressed.

## Conclusion

This study provides initial evidence that certain conditions or diseases may be less successful in medical crowdfunding based on perceived features of worthiness, such as is the case of abortion. This is an area of concern in medical crowdfunding – that certain campaigns will not be funded equitably. This is an important finding as GoFundMe positions itself as an effective and useful tool to help persons raise money for medical reasons. While the GoFundMe crowdfunding platform has an admirable mission and is a useful stop-gap for many persons to access healthcare, issues such as this need to be acknowledged and subsequently addressed.

## Data Availability

The data used for the current study are available from the corresponding author on reasonable request.

## Abbreviations

HIV/AIDS: Human immunodeficiency virus / acquired immunodeficiency syndrome
USD: United States dollars
